# Taiwanese Political Parties can be Categorized by Face, by Those Who Reported Making Face-To-Trait Inferences

**DOI:** 10.3389/fpsyg.2015.01931

**Published:** 2016-01-11

**Authors:** Shun-Fu Hu, Chien-Kai Chang, Yu-Chen Chen, Sarina Hui-Lin Chien

**Affiliations:** ^1^Graduate Institute of Neural and Cognitive Sciences, China Medical UniversityTaichung, Taiwan; ^2^Department of Chinese Medicine, China Medical UniversityTaichung, Taiwan

**Keywords:** face perception, political membership, face-to-trait inference, spontaneous trait-inference, external contour

## Abstract

The present study aims to replicate and extend Rule and Ambady (2010a)'s findings that Republicans and Democrats could be differentiated by face. In Experiment 1, undergraduates categorized 50 gray-scale full-face photos of candidates of the two major political parties in Taiwan, the Kuomingtang (KMT) and the Democratic Progressive Party (DPP). Using identical stimuli and procedure, Experiment 2 tested 25- to 57-year-olds. Experiment 3 tested undergraduates with cropped photos, Experiment 4, with photos devoid of the mouth and chin area. At the end of each Experiment, we interviewed the participants about the strategies used. Results showed that undergraduates could categorize KMT and DPP with accuracies significantly higher than chance in full-face photos (Experiment 1), *M* = 0.524, *p* = 0.045, cropped photos (Experiment 3), *M* = 0.534, *p* = 0.016, and photos devoid of the mouth-and-chin area (Experiment 4), *M* = 0.530, *p* = 0.048. Adults aged between 25 and 57 could also categorize full-face photos (Experiment 2), *M* = 0.557, *p* < 0.001. Analysis on strategy use revealed that the better-than-chance performance may be a unique contribution of those who reported making face-to-trait inferences. In sum, we replicated Rule and Ambady's (2010a) results in East Asian and found that face-to-trait inferences may be essential.

## Introduction

People automatically categorize others (Macrae and Bodenhausen, 2000). In just a glance at faces, people can effortlessly identify perceptually salient features such as age, gender, and emotions, with relatively little information (Gosselin and Schyns, 2001, 2004). Moreover, recent studies revealed that people are even capable of guessing, with an accuracy significantly higher than chance, the membership of perceptually ambiguous social groups (Rule et al., 2009), such as sexual orientation (Rule and Ambady, 2008), religion (Rule et al., 2010c), and political affiliation (Rule and Ambady, 2010a), which is the interest of the present study.

Rule and Ambady (2010a) not only demonstrated that North American university students could differentiate Democrats from Republicans by face photos, but also very likely, that such categorization was achieved via spontaneous trait-inference (STI; Willis and Todorov, 2006) or face-to-trait inference (FTI), a mechanism by which one extrapolates parameters on a face and assign personality traits to them (Willis and Todorov, 2006). For instance, “babyfacedness” tends to be interpreted as likable, trustworthy, yet incompetent (Berry and McArthur, 1985; Poutvaara et al., 2009). Showing that a more “powerful” face was more likely identified as “Republican” and a face with more “warmth” was more suggestive of a “Democrat,” Rule and Ambady (2010a) suggested that the ability to identify political membership may have derived from face-to-trait inferences that are congruent with the Republican and Democrat stereotypes.

Thus far, previous studies have suggested that face-to-trait inferences are ubiquitous and fast (Todorov and Uleman, 2002, 2003; Willis and Todorov, 2006), consensual across cultures (Rule et al., 2010b), may be consequential, for instance, in electoral outcome (Todorov et al., 2005; Ballew and Todorov, 2007; Rule et al., 2010b), and may have an early ontogeny, at about 3 or 4 years of age (Cogsdill et al., 2014). However, just to what extent the STI or FTI pertain to the ability to categorize perceptually ambiguous social groups has been relatively unexplored. Moreover, it is still subjected to further investigation as to whether an individual's age and voting experience serve to enhance the performance on the membership categorization task.

Arguably similar to the U.S., Taiwan is also a bipartisan democracy, with two dominant parties Democratic Progressive Party (DPP) and Kuomintang (KMT, sometimes referred to as the Chinese Nationalist Party; the two parties listed in alphabetical order). This makes Taiwan a suitable vehicle to test the cross-cultural generality of Rule and Ambady (2010a)'s results. The present study comprises four Experiments. Experiment 1 adopted gray-scale full-face photos of current political candidates to test Taiwanese university students' ability to categorize DPPs and KMTs by face. With precisely the same stimuli and procedure, Experiment 2 recruited Taiwanese adults aged between 25 and 57 to explore the roles of age and voting experience in the performance on the same task. Based on the first two experiments, we made preliminary attempts to explore the relationship between participants' strategies of guessing and their accuracies, and observed that participants who solely relied on observable features, such as hairstyle and dress formality, to categorize photos, tended to be worse guessers than those who reported making face-to-trait inferences. Thus, we designed Experiment 3, where we cropped the face to drastically reduce the information of the hairstyle and the shape of the face. Equally from the results of the inchoate attempts, smile as a criterion to categorize DPP and KMT correlated negatively with response accuracy. Experiment 4 hence utilized photos of faces devoid of the mouth-and-chin area. Both Experiments 3 and 4 recruited separate groups of university students. In all the four Experiments, we interviewed each participant with the same open question “How did you guess?” after he or she completed all the experimental trials. This was to gain insight about the nature and effectiveness of the strategies.

## Methods and materials

### Experiment 1

#### Participants

A total of 38 (20 females) non-politically affiliated undergraduates, aged between 20 and 24, joined the study. They are primarily students at China Medical University, Taichung, Taiwan. Informed consent was obtained prior to the experiment. All participants had normal or corrected-to- normal vision (20/20); they were naïve to the purposes of the experiment, and were tested individually in a quiet, moderately lit room. After completing a randomized block of 50 test trials, each participant received a gift certificate or cash for their participation. Three participants were excluded due to a high number of candidates (more than 8) they had known before the experiment. The criterion was set correspondent to the 95th percentile rank of the total numbers of recognition across all the four experiments. The final data set consisted of 35 participants (18 females) with an average age of 21.143. All the experiments in the present research adhered to the humanitarian concerns proposed in the Declaration of Helsinki, and was approved by the local Institutional Review Board.

#### Apparatus and stimuli

**Figure 1:**
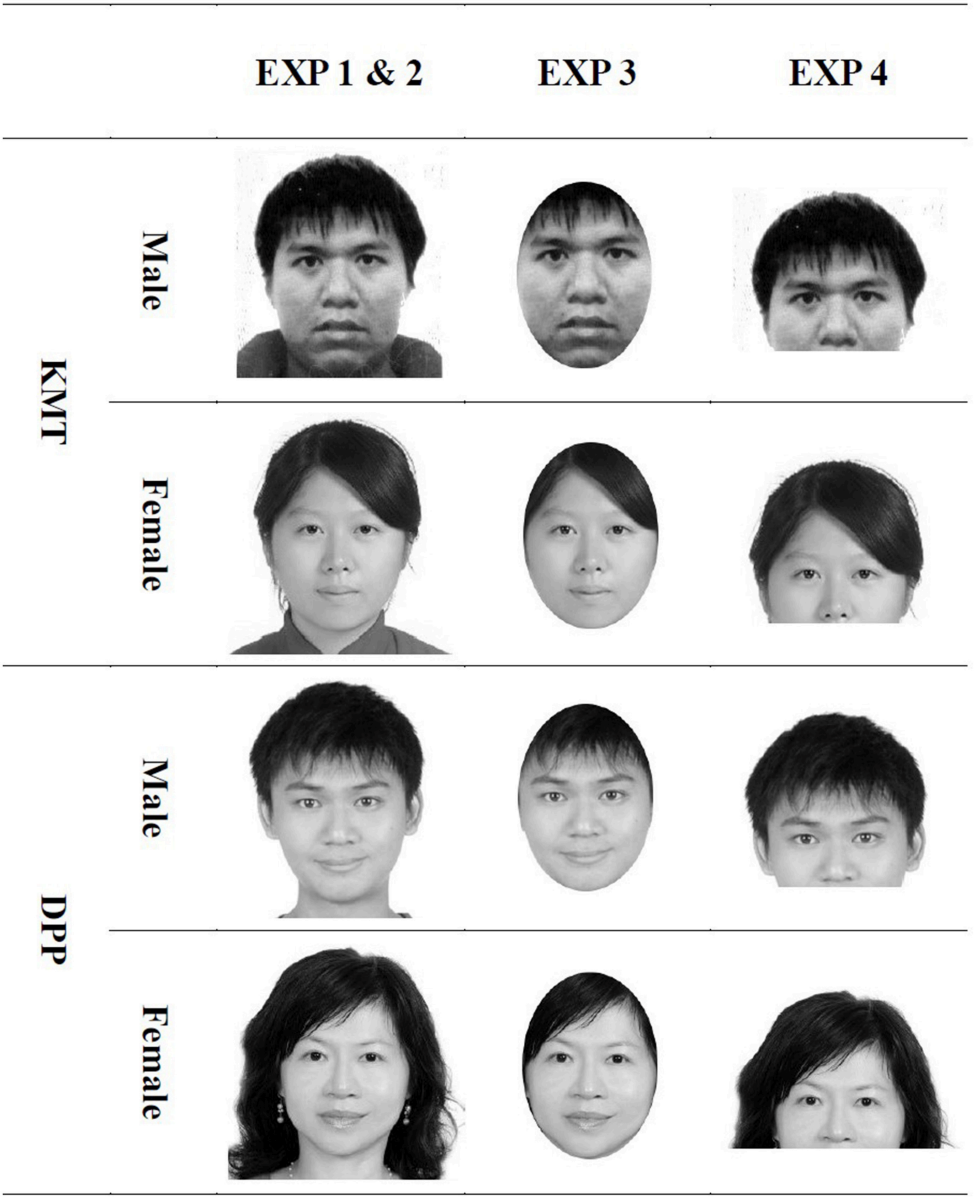
**The sample face stimuli used in the four experiments**. Experiments 1 and 2 uses full-face photos, Experiment 3 oval-cropped, and Experiment 4, photos devoid of the mouth-and-chin area. The images shown here do not represent actual candidates of KMT and DPP because of the concern of copyright permission.

A desktop computer (Acer Veriton M460) with 22″ LCD monitor (Chimei CMV 221) and E-Prime Professional 2.0 (Psychological Software Tools, Sharpsburg, PA) were used to run the experiment. The participants were seated on chairs adjusted to heights such that their eyes could fixate on the center of the screen, at a distant of ~57 cm. The stimuli consisted of 50 gray-scale photos of current KMT and DPP members, each 25 photos, accessed from the website of the 2012 Taiwanese Legislative Election by the Central Election Commission (http://web.cec.gov.tw). Each selected photo was cropped and resized proportionally to 21 cm (width) by 17 cm (height) on the monitor display occupying ~21° by 17° in visual angle at a viewing distance of 57 cm. The resolution was 96 dpi. Figure 1 illustrates the sample photos of the candidates in the four experiments.

Photos of candidates with extensive media coverage were not included; the number of candidates with glasses in both parties was approximately equal. To control for gender ratio, there were 5 female photos and 20 male photos in both parties. The ratio, 1–4, was close to the base rates of the populations of both parties. To control for the perceived age of the candidates in the photos, an additional rating study with pen-and-paper questionnaires was conducted. With informed consent, the 15 participants (8 females), whose age ranged from 18 to 53 years (*M* = 27.667, *SD* = 8.807), reported their perception of the age of each of the 50 photos that were used in our experiments. The participants did so by categorizing the photos into the five given age ranges, 20–29, 30–39, 40–49, 50–59, and 60–69, and their answers were then converted into medians of each age range, i.e., 24.5, 34.5, 44.5, 54.5, and 64.5. Although a paired *t*-test revealed a significant difference between the perceived age of DPP (*M* = 46.524, *SD* = 7.573) and KMT candidates (*M* = 48.607, *SD* = 6.927), *t*_(14)_ = 5.461, *p* < 0.001, Cohen's *d* = 1.460, a 2-year age difference at the age range of 40s was unlikely to be consequential.

#### Procedure

**Figure 2:**
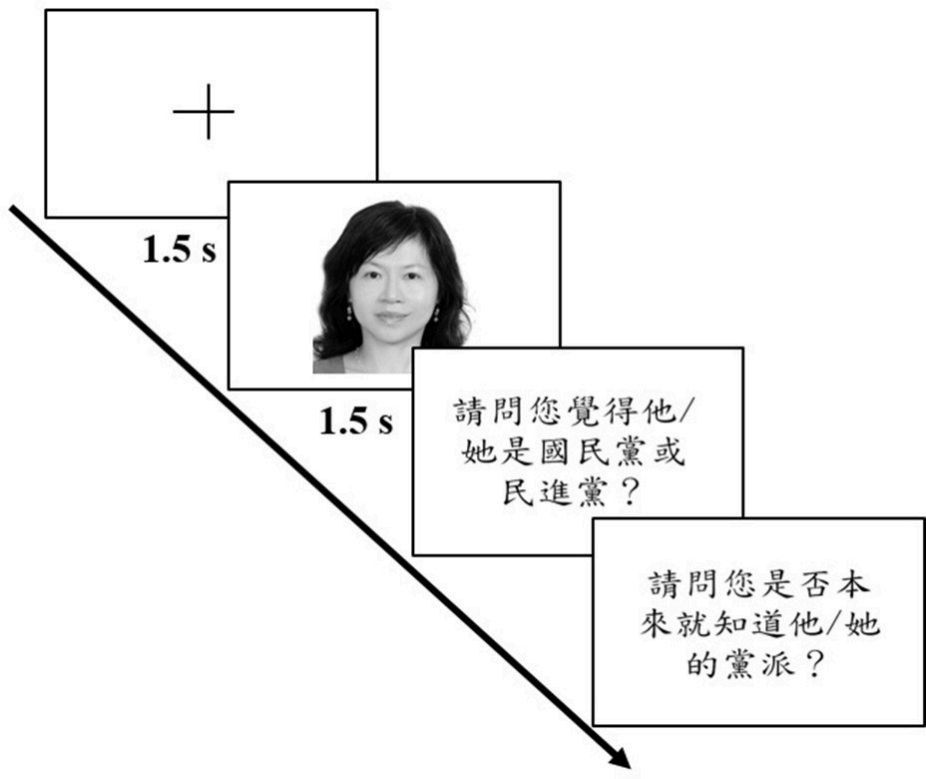
**Illustration of a sample trial for all four experiments**. The translation of the first question is “Which political party do you think he/she belongs to, KMT or DPP?” and that of the second question is “Do you already know his/her political party?”

Participants were asked to categorize a total of 50 photos of candidates as either DPP or KMT members. Figure 2 illustrates the sequence of a trial: a fixation cross for 1.5 s, a photo of either a DPP or a KMT member for another 1.5 s, and the two subsequent questions: “Which political party do you think he/she belongs to, KMT or DPP?” and “Do you already know his/her political party?” Not until the first question was answered by key press would the second question be displayed, and not until the second question was responded to would the program proceed on to the next trial. Upon completion, a smiley face would appear. Prior to the experiment, four practice trials, of four famous political candidates two for each party, were given to participants to ensure that they understood the task. The entire procedure took ~15 min for a participant to finish. After finishing all the 50 trials, participants were interviewed by one of the experimenters and were asked the open question “How did you guess?” The average number of trials of candidates of whom the participants reported to have known the political party was 1.20, and the median was 0. These trials were to be excluded in subsequent analyses.

### Experiment 2

#### Participants

A total of 37 non-politically-affiliated adults (20 females) whose age ranged from 25 to 57 were recruited from an online forum and by Internet advertisements. Prior to the experiment, the participants completed a written informed consent, and a form surveying their age, profession, the years of education, and the numbers of elections on which they voted since 1996. They were primarily citizens of the Taichung Metropolitan Area, Taiwan. All participants had normal or corrected-to- normal vision (20/20); they were naïve to the purposes of the experiment, and were tested individually in a quiet, moderately lit room. Each participant received a gift certificate or cash for their participation. Three participants were later excluded due to self-reported prosopagnosia (1), or a high number of trials on which they indicated recognition (2). The final data set consisted of 34 participants (18 females), their average age 35.912 years. Table 1 summarizes the age, years of education, voting experience, and the number of participants of each experiment.

**Table 1:** **Characteristics of the participants**.

	**EXP1**	**EXP2**	**EXP3**	**EXP4**
Numbers of participants	35 (18)	34 (18)	35 (21)	41 (19)
Mean age	21.143 (1.004)	35.912 (7.45)	19.371 (1.031)	20.732 (1.119)
Mean number of votes	N/A	3.938 (1.865)	N/A	N/A
Mean years of education	15.143 (1.004)	15.750 (1.867)	13.371 (1.031)	14.732 (1.119)

*The numbers in parentheses in the first row (Numbers of participants) indicate the number of female participants. The numbers in parentheses in the 2nd, 3rd, and the 4th rows indicate standard deviations*.

#### Apparatus, stimuli, and procedure

The apparatus and procedure were the same as in Experiment 1. The average number of trials of candidates of whom participants reported to have known the political party was 1.91, and the median was 1. These trials were to be excluded in subsequent analyses.

### Experiment 3

#### Participants

Another group of 41 (25 females) non-politically affiliated undergraduates, with an age range from 19 to 23 were recruited. Informed consent was obtained prior to the experiment. All participants had normal or corrected-to- normal vision (20/20); they were naïve to the purposes of the experiment, and were tested individually in a quiet, moderately lit room. After completing a randomized block of 50 test trials, each participant received a gift certificate or cash for their participation. Six participants were excluded due to procedural errors (2) or for knowing more than eight candidates (4). The final data set consisted of 35 participants (21 females), with a mean age of 19.371 years.

#### Apparatus and stimuli

The apparatus was the same as in Experiment 1. The stimuli were oval-cropped versions of exactly the same candidates selected in the Experiments 1 and 2. As shown in Figure 1, each photo was cropped by Photoimpact 10 (Ulead, Taipei) in a way that the parts of image within an elliptic area of 220 (width) by 160 (height) pixels were retained while the parts that fell out were deleted, and that the chin of the candidate was moved to mark the lowest boundary of the elliptic area. By doing so, the observable information about the hairstyle and the shape of the face were drastically reduced. When displayed on the screen, the cropped photos extended about 12 cm horizontally and 13 cm vertically.

#### Procedure

The procedure was identical to that in Experiment 1. The average number of trials of candidates of whom participants reported to have known the political party was 1.14, and the median was 0. These trials were to be excluded in subsequent analyses.

### Experiment 4

#### Participants

We recruited another group of 43 non-politically affiliated undergraduates (19 females). Informed consent was obtained prior to the experiment. All participants had normal or corrected-to- normal vision (20/20); they were naïve to the purposes of the experiment, and were tested individually in a quiet, moderately lit room. After completing a randomized block of 50 test trials, each participant received a gift certificate or cash for their participation. Two participants were excluded because of misunderstanding the task (1) or recognition of over 8 candidates (1). The final data set hence consisted of 41 participants (19 females), with a mean age of 20.732 years.

#### Apparatus and stimuli

The apparatus was the same as in Experiment 1. The stimuli were the same candidates selected in the previous experiments but the mouth and chin area of the face was removed using Ulead Photoimpact 10, as in Figure 1. The size of each image was 260 pixels (width) by 200 pixels (height), 21 cm (width) by 12.1 cm (height) when displayed on the screen. Each photo occupied ~21° in visual angle and had a resolution of 96 dpi.

#### Procedure

Experiment 4 followed the same procedure as in Experiment 1. The average number of trials of candidates of whom participants reported to have known the political party was 2.17, and the median was 1. These trials were to be excluded in subsequent analyses.

## Results

### Experiment 1

**Figure 3:**
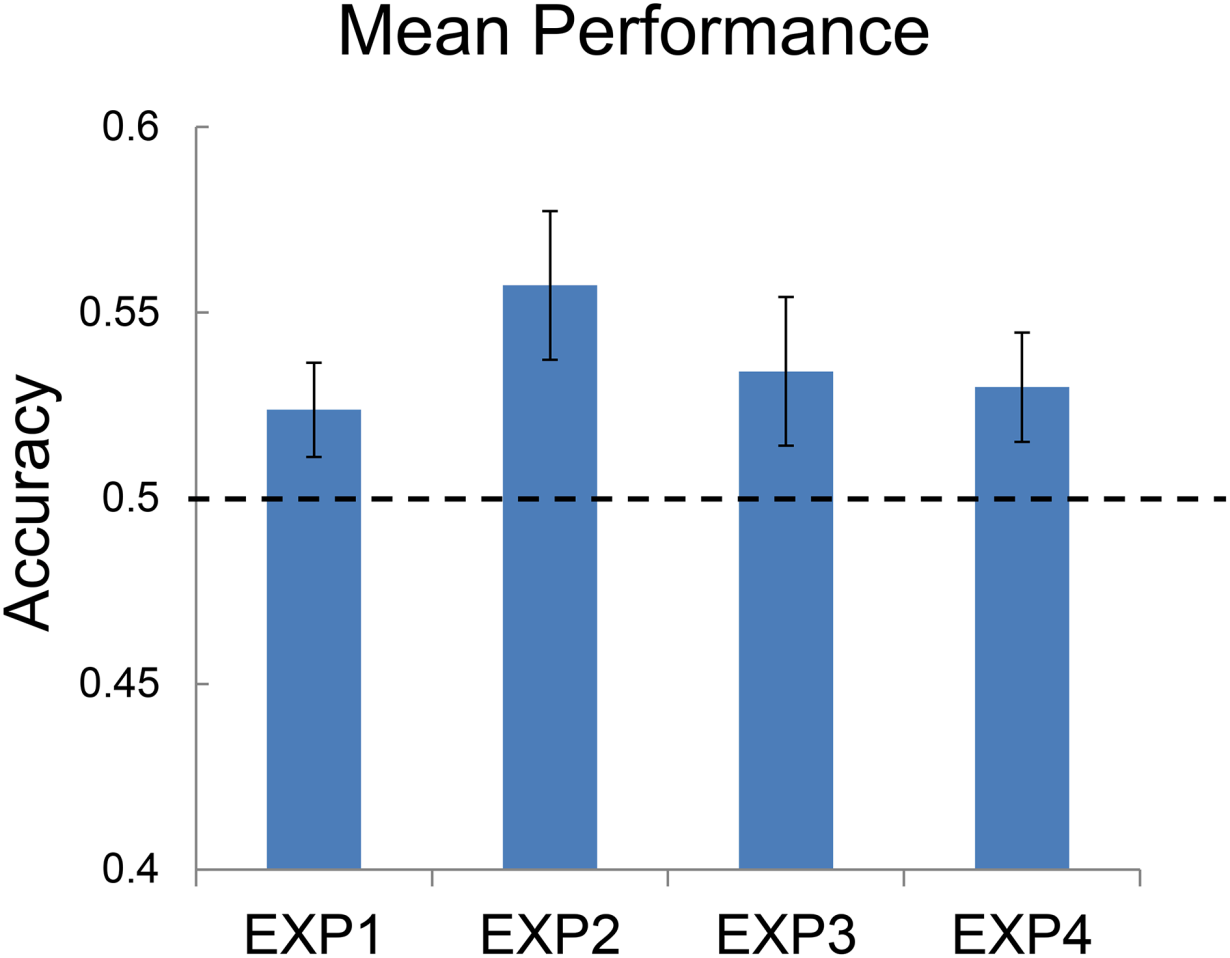
**Group mean accuracies of categorizations in the four experiments**.

The accuracy of categorizations of each participant was measured by percentage correct of the items he/she did not indicate recognition. Sensitivity measure *d*′ and response bias *c* were calculated based on the hit rates of and false alarms of KMT items, though changing it to DPP would not have affected *d*′ or *c*. Another accuracy and sensitivity measure *A*′, which also took hits and false alarms into account, was also calculated. Since *A'*s across the four experiments behaved in a similar to percentages correct (mean accuracies), the present study reports only mean accuracies. Figure 3 illustrates the group mean accuracies for all four experiments.

In Experiment 1, the undergraduate participants were able to categorize KMT and DPP candidates significantly better than chance. The mean accuracy (*M* = 0.524, *SD* = 0.068) was significantly different from chance, *t*_(34)_ = 2.075, *p* = 0.045, Cohen's *d* = 0.351. Based on Signal Detection Theory, the hit rate based on KMT was 0.547, the false alarm rate 0.499, yielding a *d*′ = 0.129 (*SD* = 0.036). The response bias *c* was 0.065 (*SD* = 0.209), indicating a slight bias toward categorizing photos as KMT. Another sensitivity and accuracy measure *A*′ = 0.519 (*SD* = 0.052), which takes into account false alarms and hits, was also significantly different from 0.50, *t*_(34)_ = 2.172, *p* = 0.037, Cohen's *d* = 0.367. None of these measures differed according to the gender of participants.

**Figure 4:**
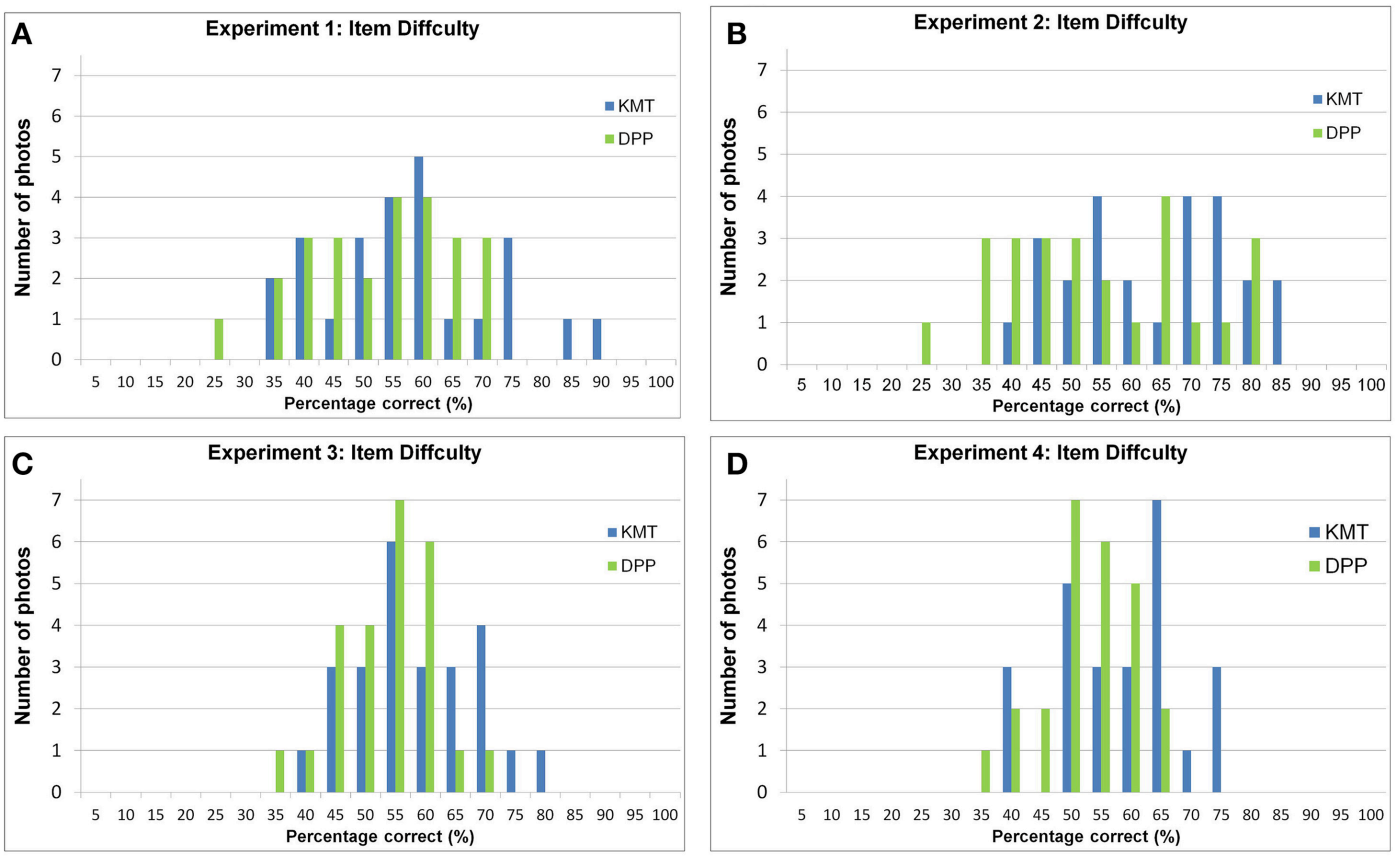
**The distribution of difficulties, measured by the percentage correct among participants who correctly categorized the item, in Experiments 1(A), 2(B), 3(C), and 4(D)**. The abscissa shows the difficulties of the items in a reversed manner; the ordinate denotes the number of items.

To ensure that the aforementioned results were not attributable to extremely easily categorized photos that biased the overall accuracy upwards or extremely difficult items, we further analyzed the distribution of item difficulties, as shown in Figure 4A. The difficulty of an item was defined as the probability, in percentage correct, of it correctly categorized by participants who had not known to which party it belonged prior to the experiments; the higher the difficulty, therefore, the easier the item. The abscissa of Figure 4A represents the item difficulties at a 5% bin, from 0%, signifying extreme difficulty, to 100%, indicating perfect categorizability. The ordinate indicates the number of such items. One can observe that (1) the percentage correct of items ranged from 25 to 90%, (2) none of the items could be neither 100% correctly categorized nor 0%, and that (3) the item difficulties were normally distributed, confirmed by the test of normativity Shapiro–Wilk *W*_(49)_ = 0.979, *p* = 0.521.

### Experiment 2

As in Experiment 1, the adult participants aged between 25 and 57 were able to categorize KMT and DPP candidates significantly better than chance. The mean accuracy (*M* = 0.557, *SD* = 0.074) was significantly greater than chance, *t*_(33)_ = 4.514, *p* < 0.001, Cohen's *d* = 0.774. According to the Signal Detection Theory, the hit rate based on KMT was 0.609, the false alarm rate 0.494, yielding a *d*′ = 0.304 (*SD* = 0.395). The response bias *c* was 0.135 (*SD* = 0.178), indicating a bias toward categorizing photos as KMT; another sensitivity and accuracy measure, *A*′ = 0.544 (*SD* = 0.056), which takes into account false alarms and hits, was also significantly different from 0.50, *t*_(33)_ = 4.559, *p* < 0.001, Cohen's *d* = 0.781. None of these measures differed according to the gender of participants.

Among the participants in Experiment 2, age showed a slightly negative correlation with accuracy (*r* = −0.180), so did the number of votes (*r* = −0.220); on the other hand, years of education (*r* = 0.212) slightly positively correlated with categorization success. However, none of the correlations were significant.

As demonstrated in Figure 4B, as in Experiment 1, (1) the percentage correct of items ranged from 25 to 85%, (2) none of the items could be neither 100% correctly categorized nor 0% in Experiment 2, and that (3) the item difficulties were normally distributed, confirmed by the test of normativity Shapiro–Wilk *W*_(49)_ = 0.971, *p* = 0.265.

### Experiment 3

As in Experiments 1 and 2, the undergraduate participants in Experiment 3 were able to categorize KMT and DPP candidates significantly better than chance, even when the external contours of the photos were removed. The mean accuracy (*M* = 0.534, *SD* = 0.080) was significantly different from chance, *t*_(34)_ = 2.528, *p* = 0.016, Cohen's *d* = 0.427. The hit rate based on KMT was 0.559, the false alarm rate 0.490, yielding a *d*′ = 0.180 (*SD* = 0.420). The response bias *c* was 0.065 (*SD* = 0.196), indicating a slight bias toward categorizing photos as KMT; another sensitivity and accuracy measure, *A*′ = 0.524 (*SD* = 0.057), which takes into account false alarms and hits, was also significantly different from 0.50, *t*_(34)_ = 2.525, *p* = 0.016, Cohen's *d* = 0.427. None of these measurements differed according to the gender of participants.

As demonstrated in Figure 4C, it can be observed that (1) the percentage correct of items ranged from 35 to 80%, (2) none of the items could be neither 100% correctly categorized nor 0% in Experiment 2, and that (3) the item difficulties were normally distributed, confirmed by the test of normativity Shapiro–Wilk *W*_(49)_ = 0.982, *p* = 0.672.

### Experiment 4

As in Experiments 1, 2, and 3, the undergraduate participants in Experiment 4 were able to categorize KMT and DPP candidates significantly better than chance. The mean accuracy (*M* = 0.530, *SD* = 0.094) was significantly different from chance, *t*_(40)_ = 2.032, *p* = 0.048, Cohen's *d* = 0.317. According to Signal Detection Theory, the hit rate based on KMT was 0.561, the false alarm rate 0.500, yielding a *d*′ = 0.164 (*SD* = 0.502). The response bias *c* was 0.083, indicating a slight bias toward categorizing photos as KMT; another sensitivity and accuracy measure, *A*′ = 0.525 (*SD* = 0.072), which takes into account false alarms and hits, was also significantly different from 0.50, *t*_(40)_ = 2.179, *p* = 0.035. Cohen's *d* = 0.340. None of these measurements differed according to the gender of participants.

Figure 4D demonstrates that (1) the percentage correct of items ranged from 35 to 80%, (2) none of the items could be neither 100% correctly categorized nor 0% in Experiment 2, and that (3) the item difficulties were normally distributed, confirmed by the test of normativity Shapiro–Wilk *W*_(49)_ = 0.958, *p* = 0.079.

### Comparing the four experiments

To look into the effect of the Manipulations on photos, an ANOVA was conducted to compare the performance of undergraduate participants in Experiments 1, 3, and 4, with mean accuracies as the dependent variable. No significant main effect of the Manipulations on photos was found. Notwithstanding, in all the four experiments, the participants' mean accuracies, and *A'*s, significantly differed from 0.50, all with a small to medium-high effect size. For Taiwanese undergraduate students, KMT and DPP candidates could be differentiated based on full-face photos (Experiment 1), oval-cropped photos (Experiment 3), and even photos without mouth-and-chin areas (Experiment 4). For Adults aged between 25 and 57 (Experiment 2), KMT and DPP could also be told apart at least based on full-face gray-scale photos, but they did not perform differently from university students (Experiment 1) even if they were older and had more voting experience. In all the 4 experiments, participants had a slight tendency toward categorizing photos as KMT candidates. Notwithstanding, all their *A'*s, which took hits and false alarms into account, were still all significantly greater than 0.50.

As demonstrated in Figure 4, none of the photos were perfectly categorized nor misplaced in all the Experiments. The item difficulties of one experiment correlated significantly with the other three [*r*_(14)_ = 0.349, *p* = 0.014; *r*_(24)_ = 0.556, *p* = 0.000; *r*_(34)_ = 0.288, *p* = 0.044; *r*_(23)_ = 0.387, *p* = 0.006; *r*_(24)_ = 0.556, *p* = 0.000; *r*_(34)_ = 0.288, *p* = 0.044], suggesting that candidates' photos that were difficult to correctly categorize in one experiment were also likely difficult in the other three experiments, and *vice versa*.

### Analysis of strategy

In the preliminary analyses, we listed a number of common strategies among the four Experiments, and coded them based on the *presence* (=1) or *absence* (=0) of the use of each strategy. Though none of the point-biserial correlations between the use of these strategies across participants and their categorization accuracy were significant, we observed that strategies that pertain only to observable features or demographic information tended to correlate negatively with accuracy, while face-to-trait inferences tended to correlate positively with accuracy. An example of a clear “observable-feature” was “KMTs tend to have slicked hair; DPPs tend to have smaller faces.” This observation inspired Experiment 3, which encourages the focus on the interior parts of the face to make face-to-trait inferences, and Experiment 4, which discourages using smile as a categorizing criterion.

With the knowledge of Rule and Ambady (2010a)'s proposal that the power-and-warmth division may enable categorization success, we initially profiled and quantified participants' self-reported strategy use. However, for Taiwanese participants, power-and-warmth division did not predict good performance for categorizing KMT and DPP. Rather, interesting results unfolded as we classified participants' into three mutually exclusive strategy groups based on the tendency to make face-to-trait inferences: “Face-to-trait inferences for both parties,” (Group 1) “Face-to-trait inferences for either party,” (Group 2), and “No face-to-trait inference” (Group 3) (i.e., relying on observable features or demographic information only). A typical self-report that would render the participant categorized as Group 1 was “KMTs look like powerful business people, and DPPs look honest grass-root scholars,” a Group 2 answer would be “DPPs look aggressive (face-to-trait), and KMTs tend to be elder (not face-to-trait),” and a Group 3 answer would be “KMTs tend to have a larger face, and females are more likely DPPs.”

Table 2 shows frequency counts of the participants of the three strategy groups across the four experiments. Out of the 35 participants in Experiment 1, 9 (25.71%) fell into Group 1, 12 (34.29%), Group 2, and 14 (40%), Group 3. Out of the 34 participants in Experiment 2, 17 (50%), 11 (32.35%), and 6 (17.14%) belonged to Group 1, 2, and 3, respectively. With a total of 35 participants, Experiment 3 saw 16 (45.71%), 13 (37.14%), and 6 (17.14%), in Group 1, 2, and 3, respectively. Experiment 4, among the 41 participants, had 10 (24.39%) in Group1, 13 (31.71%) in Group 2, and 18 (43.90%) in Group 3.

**Table 2:** **The frequency counts of participants that fell into the three strategy groups in the four experiments**.

	**Group 1**	**Group 2**	**Group 3**	**Total**
EXP1	9	12	14	35
EXP2	17	11	6	34
EXP3	16	13	6	35
EXP4	10	13	18	41
Total	52	49	44	145

*Group 1 refers to participants who made face-to-trait inferences for both parties, Group 2, for either party, and Group 3, for neither*.

The Chi-square tests of independence revealed that the distribution of strategy use was associated with the manipulations of the four experiments, χ^2^ = 12.920, *df* = 6, *p* = 0.044. Cramer's *V* = 0.211. More participants in Experiment 2 (adults aged between 25 and 57) seemed to fall into Group 1, and less into Group 3, than did their university students counterparts (Experiment 1). Chi-square test revealed marginal significance, χ^2^ = 5.692, *df* = 2, *p* = 0.058, Cramer's *V* = 0.287. In Experiment 3, cropping the face appeared to have encouraged participants to make face-to-trait inference (more Group1 and less Group 3) compared with Experiment 1, χ^2^ = 5.200, *df* = 2, *p* = 0.074, which was also marginally significant, Cramer's *V* = 0.272. Experiment 4, however, had a similar distribution of strategy use to Experiment 1.

Table 3 lists the mean accuracies of the participants of the three strategy groups across the four experiments. We conducted a Two-way between-subject ANOVA with Manipulations on photos (Experiments 1, 3, and 4) and Strategy Groups (1, 2, and 3) as the two between-subject factors, while the dependent variable was the accuracy of categorizations. The results showed a significant main effect of Strategy Groups, *F*_(2, 102)_ = 4.117, *p* = 0.002; ${\eta_{p}}^{2}=0.075$; the main effect of the Manipulations on photos was not significant, nor was the interaction effect. *Post Hoc* comparisons using Scheffé's method indicated that participants that reported making face-to-trait inferences for both parties (Group 1, *M* = 0.563, *SE* = 0.064) had a significantly higher accuracy than those who only inferred for one party (Group 2, *M* = 0.513, *SE* = 0.086), *p* = 0.031, or those who did not at all (Group 3, *M* = 0.515, *SE* = 0.084), *p* = 0.041. Group 2 and 3 did not differ from each other. In addition, another Two-way ANOVA comparing the performance of participants of Experiments 1 and 2 and strategy groups yielded comparable results: Group (Experiments 1, and 2) had no significant main effect, while Strategy Groups did, *F*_(2, 63)_ = 5.936, *p* = 0.004; ${\eta_{p}}^{2}=0.159$. Similarly, Scheffé's method indicated Group 1, *M* = 0.578, *SE* = 0.082, had a marginally higher accuracy than Group 2, *M* = 0.531, *SE* = 0.057, *p* = 0.061, and a significantly higher accuracy than Group 3, *M* = 0.502, *SE* = 0.051, *p* = 0.002. The latter two groups did not differ from each other.

**Table 3:** **The mean accuracies and standard deviations (in parentheses) of participants that fell into the three strategy groups in the four experiments**.

	**Group 1**	**Group 2**	**Group 3**	**Mean accuracy**
EXP1	0.567 (0.077)	0.518 (0.059)	0.501 (0.059)	0.524 (0.068)
EXP2	0.583 (0.087)	0.546 (0.053)	0.506 (0.028)	0.557 (0.074)
EXP3	0.561 (0.060)	0.502 (0.091)	0.534 (0.089)	0.534 (0.080)
EXP4	0.564 (0.065)	0.518 (0.105)	0.519 (0.100)	0.530 (0.094)
Mean	0.570 (0.072)	0.520 (0.080)	0.514 (0.079)	0.536 (0.080)

*Group 1 refers to participants who made face-to-trait inferences for both parties, Group 2, for either party, and Group 3, for neither*.

## Discussion

With the face categorization task and an open interview at the end, the present study tested the cross-cultural generality of Rule and Ambady's results (2010a) in Taiwan, and the roles of age, face-to-trait inferences, external contour and mouth-and-chin area of the faces. We demonstrated that non-politically-affiliated Taiwanese University students (Experiment 1), as well as adults aged between 25 and 57 (Experiment 2), were able to categorize KMT and DPP candidates significantly better than chance, based on full-face photos. We further discovered that the capacity to categorize DPP and KMT was robust across experimental manipulations: reducing information from the exterior contour (Experiment 3) or removing the mouth-and-chin area of the face (Experiment 4) did not impede the performance. Shown in Table 3, the analysis of strategies further revealed that those who reported making face-to-trait inferences for both parties (Group 1) had significantly higher accuracies than did the other two groups. Further, the better-than-chance performance on the categorization task seemed a unique contribution of Group 1, across the four experiments; Group 2's and Group 3's accuracy did not differ from chance.

A key difference in methodology allowed the present study and Rule and Ambady (2010a)'s to explore different aspects of the effect of age: instead of “rejuvenating” the photos to those in college yearbooks as Rule and Ambady did (Study 2), we “geronticized” the participants by recruiting a group of adults aged between 25 and 57, to directly measure the effect of participants' age (and voting experience, Experiment 2). Rule and Ambady (2010a) discovered robust categorizability even with photos of young candidates; we found unchanged performance across two age groups with a cut point of 25 years of age. However, the 34 participants in Experiment 2 encompassed a rather wide spectrum of age ranges. The failure to find a significant correlation between age and successful categorizations may partly be because each age range was underrepresented. Nevertheless, the fact that Experiment 2 (mean age = 35.912) had a marginally higher proportion of guessers who reported making face-to-trait inferences to both parties (Group 1, in Tables 2, 3), who generally performed better, than Experiment 1, may still reflect combined effects of age, voting experience, and other aspects of social experience not addressed in the present study.

Moreover, as shown in Figure 4, the aforementioned results were not attributable either to extremely easily categorized photos that biased the overall accuracy upwards, or to an unaccounted interaction between the manipulations and the difficulty of test trials, nor were they due to recognition. They were also unlikely due to a perceived 2-year age difference of photos or gender ratio. The small to medium-effect sizes may be acceptable, given the “perceptually ambiguous” nature of political social grouping.

The present study agrees with Macrae and Bodenhausen's (2000) idea that people think categorically of others, and indirectly confirms that spontaneous trait inferences (Willis and Todorov, 2006) were made through self-reports during participants' interviews of strategies. Most importantly, the present study replicated Rule and Ambady (2010a)'s results in Taiwan, that participants were able to categorize political parties based on photos.

As for the reason why the general tendency to report making face-to-trait inferences was associated with good performance, we would like to propose the following explanations: Like most experts, good guessers in our experiments may have developed clear (and individualized) representations that necessitated abstraction to form organizing rules with which they distinguish faces, abstraction that face-to-trait inferences helped with. A tendency toward face-to-trait inferences, hence, though may not have caused, is at least reflective of such expertise. Though the exact mechanism remains unclear, it is safe to assume that face-to-trait inferences, an ability that might have an early ontogeny (Cogsdill et al., 2014), are involved in categorizing perceptually ambiguous social groups, such as political parties.

Note that, as one of the limitations of the present study, participants who did not report making face-to-trait inferences could still have inferred personality based on photos, only less consciously or they were simply reluctant to report. However, one may still hypothesize that, those who reported extensive face-to-trait inferences were doing it, at the conscious level. Also notably, the observations of the present study did not rule out the putative contributions of face-to-trait inferences based on demographic information to accurate categorizations. The inference that “DPPs are more energetic (face-to-trait inferences),” for example, may be due to low facial maturity or a younger perceived age (demographic information), rather than the configuration of the face. The concrete message, however, was that an answer that referred only to the observable fact of age difference, such as “DPPs tend to be younger,” did not predict accuracy.

Interestingly, face-to-trait inferences may be highly individualized and variable. Participants seemed to agree on some traits while disagree on others. For example, it was highly consensual that DPPs were likely grass root Taiwanese while KMTs looked more Chinese (Mainlanders). Conversely, some perceived KMT as honest, DPP dishonest, others the reverse. Another trait on which participants disagreed was perceived abrasiveness. More importantly, those who held completely opposite opinions of the two parties could be equally precise, as long as the process of abstraction, as mentioned in the previous paragraph, was allowed to occur.

To explore the issue of why removing the external contour of the face did not jeopardize the ability to guess, one must understand the different roles of external and internal parts of the face might play. Though both have been found to be important for identity, internal parts, especially eyes, mouth, and even eyebrows, seemed to be sufficient for one to identify familiar faces (Sinha, 2002; Sadr et al., 2003). Further, an fMRI study by Chen and Tseng (2008) revealed that the external contour and the internal parts of the face may have different neural correlates. Viewing a graphically modified image of a face with only the external contour, participants had a higher level of activation in their anterior Fusiform Face Area (FFA) than when seeing the inverted version. The internal parts were more associated with the Orbital Face Area (OFA) activation. The anterior temporal Face Area, found to be more linked with the external contour, has been proposed to be the interface of face recognition and memory of faces (Collins and Olson, 2014). Taken together, the external contour of the face may serve to help retrieve memory of faces, rather than directly help with identification. Given that the task demands of the present study had more to do with categorization than identification, it is reasonable that removing the external parts of the face had no pronounced negative consequence in performance.

We originally predicted that removing the mouth-and-chin would enhance performance because the mouth and chin areas are expressive of emotions, and emotions were discovered to affect face-to-trait inferences (Knutson, 1996). In our case, “smile,” as mentioned in previous footnotes, may have confounded with face-to-trait inferences by giving an impression of “warmth,” a quality slightly more stereotypical of DPP though with disagreements. KMTs candidates that smiled, and DPPs that did not, could have been misleading to the participants. Though occasionally participants in Experiment 4 still referred to the eyes to seek evidence of smile and warmth, removing a large part of smile by deleting and the mouth and chin area, should still increase accuracy. Though this was not the case, we may at least reason that the upper part of the face, containing the major informative features such as eyes, eyebrows, and nose, seemed sufficient for the participants to categorize political parties.

In sum, the present study points to the generality of Rule and Ambady's (2010a) results in East Asia. The two dominant Taiwanese political parties, DPP and KMT, can be categorized by face with accuracy significantly higher than chance. This ability is not jeopardized by reducing information from the exterior contour or the mouth-and-chin area of the face, and is not enhanced with age or voting experience. It may necessitate face-to-trait inferences, which may be highly individualized. Future studies are needed to delve into the exact mechanisms by which such inferences help with categorizing perceptually ambiguous social groups, as represented by political affiliations.

## Author contributions

SC, SH, and YC developed the study concept. All authors contributed to the study design. Testing and data collections were performed by SH, CC, and YC. SH, and CC performed the data analysis and interpretation under the supervision of SC. SH, and SC, writed the manuscript, and SC provided critical revisions. All authors approved the final version of the manuscript for submission.

### Conflict of interest statement

The authors declare that the research was conducted in the absence of any commercial or financial relationships that could be construed as a potential conflict of interest. The reviewer Gregory West and handling editor declared their shared affiliation, and the handling editor states that the process nevertheless met the standards of a fair and objective review.

## References

[B1] Ballew C. C. Todorov A. (2007). Predicting political elections from rapid and unreflective face judgments. Proc. Natl. Acad. Sci. U.S.A. 104, 17948–17953. 10.1073/pnas.0705435104 17959769 PMC2084277

[B2] Berry D. S. McArthur L. Z. (1985). Some components and consequences of a babyface. J. Pers. Soc. Psychol. 48, 312 10.1037/0022-3514.48.2.312

[B3] Chen C.-C. Tseng R.-Y. (2008). The role of external head contours in face processing in the human occipitotemporal cortex. J. Vis. 8, 164–164. 10.1167/8.6.164

[B4] Cogsdill E. J. Todorov A. T. Spelke E. S. Banaji M. R. (2014). Inferring character from faces a developmental study. Psychol. Sci. 25, 1132–1139. 10.1177/0956797614523297 24570261 PMC5580985

[B5] Collins J. A. Olson I. R. (2014). Beyond the FFA: the role of the ventral anterior temporal lobes in face processing. Neuropsychologia 61, 65–79. 10.1016/j.neuropsychologia.2014.06.005 24937188 PMC4122611

[B6] Gosselin F. Schyns P. G. (2001). Bubbles: a technique to reveal the use of information in recognition tasks. Vision Res. 41, 2261–2271. 10.1016/S0042-6989(01)00097-9 11448718

[B7] Gosselin F. Schyns P. G. (2004). No troubles with bubbles: a reply to Murray and Gold. Vision Res. 44, 471–477. 10.1016/j.visres.2003.10.007 14680772

[B8] Knutson B. (1996). Facial expressions of emotion influence interpersonal trait inferences. J. Nonverbal Behav. 20, 165–182. 10.1007/BF02281954

[B9] Macrae C. N. Bodenhausen G. V. (2000). Social cognition: thinking categorically about others. Annu. Rev. Psychol. 51, 93–120. 10.1146/annurev.psych.51.1.93 10751966

[B10] Poutvaara P. Jordahl H. Berggren N. (2009). Faces of politicians: babyfacedness predicts inferred competence but not electoral success. J. Exp. Soc. Psychol. 45, 1132–1135. 10.1016/j.jesp.2009.06.007

[B11] Rule N. O. Ambady N. (2008). Brief exposures: male sexual orientation is accurately perceived at 50 ms. J. Exp. Soc. Psychol. 44, 1100–1105. 10.1016/j.jesp.2007.12.001

[B12] Rule N. O. Ambady N. (2010a). Democrats and republicans can be differentiated from their faces. PLoS ONE 5:e8733. 10.1371/journal.pone.0008733 20090906 PMC2807452

[B13] Rule N. O. Ambady N. Adams R. B. Jr. Ozono H. Nakashima S. Yoshikawa S.. (2010b). Polling the face: prediction and consensus across cultures. J. Pers. Soc. Psychol. 98, 1. 10.1037/a0017673 20053027

[B14] Rule N. O. Garrett J. V. Ambady N. (2010c). On the perception of religious group membership from faces. PLoS ONE 5:e14241. 10.1371/journal.pone.0014241 21151864 PMC2998417

[B15] Rule N. O. Macrae C. N. Ambady N. (2009). Ambiguous group membership is extracted automatically from faces. Psychol. Sci. 20, 441–443. 10.1111/j.1467-9280.2009.02314.x 19399971

[B16] Sadr J. Jarudi I. Sinha P. (2003). The role of eyebrows in face recognition. Perception 32, 285–293. 10.1068/p5027 12729380

[B17] Sinha P. (2002). Identifying perceptually significant features for recognizing faces, in Electronic Imaging 2002. International Society for Optics and Photonics (San Jose, CA), 12–21.

[B18] Todorov A. Mandisodza A. N. Goren A. Hall C. C. (2005). Inferences of competence from faces predict election outcomes. Science 308, 1623–1626. 10.1126/science.1110589 15947187

[B19] Todorov A. Uleman J. S. (2002). Spontaneous trait inferences are bound to actors' faces: evidence from a false recognition paradigm. J. Pers. Soc. Psychol. 83, 1051. 10.1037/0022-3514.83.5.1051 12416911

[B20] Todorov A. Uleman J. S. (2003). The efficiency of binding spontaneous trait inferences to actors' faces. J. Exp. Soc. Psychol. 39, 549–562. 10.1016/S0022-1031(03)00059-3

[B21] Willis J. Todorov A. (2006). First impressions making up your mind after a 100-ms exposure to a face. Psychol. Sci. 17, 592–598. 10.1111/j.1467-9280.2006.01750.x 16866745

